# GIS‐based G × E modeling of maize hybrids through enviromic markers engineering

**DOI:** 10.1111/nph.19951

**Published:** 2024-07-16

**Authors:** Rafael T. Resende, Alencar Xavier, Pedro Italo T. Silva, Marcela P. M. Resende, Diego Jarquin, Gustavo E. Marcatti

**Affiliations:** ^1^ Plant Breeding Sector, School of Agronomy (EA) Federal University of Goiás (UFG) Av. Esperança, s/n, Samambaia Campus Goiânia GO 74690‐900 Brazil; ^2^ TheCROP, A Precision Breeding Project Av. Esperança, n° 1533, FUNAPE, Samambaia Technological Park, Samambaia Campus – UFG Goiânia GO 74690‐612 Brazil; ^3^ Corteva Agriscience 8305 NW 62ndAve Johnston IA 50131 USA; ^4^ Purdue University 915 Mitch Daniels Blvd West Lafayette IN 47907 USA; ^5^ University of Florida 1604 McCarty Drive G052B McCarty Hall D Gainesville FL 32611 USA; ^6^ Forest Engineering Department Federal University of São João del Rei (UFSJ) Sete Lagoas Campus, MG‐424 Highway, Km 47 Sete Lagoas MG 35701‐970 Brazil

**Keywords:** ensemble modeling, geoprocessing/GIS, machine learning, phenotypic plasticity, precision breeding, random regression, reaction norms, stability and adaptability

## Abstract

- Through enviromics, precision breeding leverages innovative geotechnologies to customize crop varieties to specific environments, potentially improving both crop yield and genetic selection gains.
- In Brazil's four southernmost states, data from 183 distinct geographic field trials (also accounting for 2017–2021) covered information on 164 genotypes: 79 phenotyped maize hybrid genotypes for grain yield and their 85 nonphenotyped parents. Additionally, 1342 envirotypic covariates from weather, soil, sensor‐based, and satellite sources were collected to engineer 10 K synthetic enviromic markers via machine learning.
- Soil, radiation light, and surface temperature variations remarkably affect differential genotype yield, hinting at ecophysiological adjustments including evapotranspiration and photosynthesis. The enviromic ensemble‐based random regression model showcases superior predictive performance and efficiency compared to the baseline and kernel models, matching the best genotypes to specific geographic coordinates. Clustering analysis has identified regions that minimize genotype‐environment (G × E) interactions. These findings underscore the potential of enviromics in crafting specific parental combinations to breed new, higher‐yielding hybrid crops.
- The adequate use of envirotypic information can enhance the precision and efficiency of maize breeding by providing important inputs about the environmental factors that affect the average crop performance. Generating enviromic markers associated with grain yield can enable a better selection of hybrids for specific environments.

## Abstract 2

See also the Commentary on this article by Bowerman, **245**: 9–10.

## Introduction

The great challenge of genetics and breeding is to unravel the mechanisms involved in the phenotypic expression of a plant's genotype. Classical breeders have long told us that the phenotype results from the joint and additive action of the genotype with the environment, in addition to specific patterns generated from the interaction between them (Des Marais *et al*., 2013; Jarquin *et al*., 2014). With the availability of environmental data derived from informatics management platforms, capable of capturing detailed information about vegetation, surface structure, and terrain, now our challenge is to improve the understanding of how much the environment influences the expression of the phenotype (Resende *et al*., 2021). This knowledge makes it possible to better predict the performance of individuals in unevaluated environments.

Within environments phenotyping is particularly important in maize (*Zea mays* L.) because it is grown on most continents, in a diverse range of environmental conditions. Commercial maize cultivation typically happens in latitudes ranging from 58°N to 40°S and elevations from sea level to 3800 m above sea level (Ortiz *et al*., 2010). The development of maize hybrids consists of breeding superior genotypes for a target population of environments (TPE). Hence, maize breeding programs conduct trials that best represent the TPE. Messina *et al*. (2023) emphasize substantial advancements in maize improvement over two decades. These authors unveil a remarkable escalation in crop enhancement rates under challenging conditions, achieved by harnessing favorable alleles and incorporating physiological understanding into genetic selection methods. However, it is not known yet exactly how the maize genetics and a specific set of environmental conditions lead to observed values for yield and other agronomic traits, suggesting that predictive breeding approaches will continue to predominate (Bernardo, 2021).

The identification of superior hybrids is affected by changes in the response patterns of genotypes under different environmental *stimuli*. This phenomenon is known as genotype–by–environment (G × E) interaction. Building effective prediction models considering G × E interaction is a game‐changer for selecting hybrids with stable performance across various environmental conditions while maximizing the accuracy of recommendations for specific environments (Jarquin *et al*., 2021; Piepho, 2022). In maize, prediction model performance across various environments revealed the superiority of models integrating G × E interactions, yielding enhanced predictions for attributes such as grain yield, plant height, and ear height (Bandeira e Sousa *et al*., 2017; Cuevas *et al*., 2018; Costa‐Neto *et al*., 2021a). Furthermore, Gevartosky *et al*. (2023) demonstrate that incorporating envirotyping data into prediction models for tropical maize increases the efficiency and cost‐effectiveness of these models, enabling the allocation of resources in other stages of the breeding program.

In the evaluation of the performance of hybrids in multi‐environment trials, it is necessary to understand the wide factors influencing the target population of genotypes (TPG). The *enviromics* terminology (Resende *et al*., 2021) is herein applied in the context of precision agriculture for maize breeding. It aims to describe the envirotypes that could potentially modify the phenotypic behavior of genotypes and adaptability to different locations. Cooper & Messina (2021) further underscore the integration of enviromic technologies to enhance crop improvement by capturing detailed environmental influences on G × E interactions. Resende *et al*. (2024) further elaborated on this concept by illustrating how satellite‐enabled enviromics can provide a more precise environmental characterization, thereby refining the predictive models that are essential for optimizing genotype performance across diverse conditions. This comprehensive integration of envirotypic data is essential for accurately assessing the interaction between genotypes and environments. Ultimately, enabling the development of high‐yielding and stable maize hybrids across varied settings.

Current efforts in farming are experiencing the integration of novel precision agriculture techniques, driven by the widespread adoption of Geographic Information Systems (GIS) technologies (Annicchiarico *et al*., 2006; Lindblom *et al*., 2017; Marcatti *et al*., 2017), along with the strategic implementation of artificial intelligence (AI) methodologies for enhancing agricultural processes (Xu *et al*., 2022; Negus *et al*., 2024). Within the scope of GIS, the conventional concept of agricultural sites has transformed into discrete elements within a geographical grid, often referred to as ‘*pixels*’ or ‘*bins*’. These bins encompass various trial locations where phenotypic data is meticulously collected, while others remain devoid of such data. Consequently, while phenotypic information is accessible for a subset of these bins, AI's integration in agriculture can leverage this data for predictive efforts, facilitating informed decision‐making and fostering the development of crops with higher agricultural productivity throughout the TPE (Resende *et al*., 2024).

Under the assumption that weather stations are ubiquitous, environmental data kriging enables extrapolation to every location within the boundaries of the TPE. Remote sensing data can also be utilized, and it is freely available online (Kasampalis *et al*., 2018). Considering global climate change and anthropogenic landscape changes, knowing the cultivation area with a certain level of precision becomes essential for genotypic evaluation and recommendation (Marcatti *et al*., 2017). Environmental fluctuations influence the effectiveness of traditional selection criteria under field extreme conditions (Guadarrama‐Escobar *et al*., 2024). Genetic variation sources have been extensively studied, as the genotypic expression is closely linked to additive and nonadditive effects. Such studies can be combined with enviromics techniques to compose the different stages of a breeding cycle, considering the environment's pressure on phenotypic plasticity.

Challenges in precision agriculture data analysis are evolving alongside advancements in data availability (Wolfert *et al*., 2017). Innovations such as geotechnologies, big data, and real‐time analytics now provide reliable techniques for identifying environmental covariates, thus enhancing the success of breeding programs (Cooper *et al*., 2014; Xu, 2016; Resende *et al*., 2024). Recent studies increasingly incorporate enviromics alongside genetic data, highlighting the significance of enviromics in plant breeding, and in studies of the genotype–by–environment interactions (Crossa *et al*., 2021; Xu *et al*., 2022). Our study proposes a novel approach using random forest and mixed models to engineer enviromic markers, aiming to predict hybrid performance across observed and unobserved regions within the TPE, thereby offering detailed operational information into genotypic stability and performance across specified geographical areas.

## Materials and Methods

### Phenotypic data and geoprocessing environment

The TPG consisted of 79 hybrid genotypes of maize (*Zea mays* L.) resulting from the cross between 85 parent lines from a commercial breeding program from Corteva AgriScience®. Parental genotypes were coded as G001 to G085 and hybrids from G086 to G164. Genotypes with unknown parents are competitor hybrids. The grain yield was evaluated in 183 unbalanced field trials distributed in four Brazilian states: São Paulo (SP), Paraná (PR), Santa Catarina (SC), and Rio Grande do Sul (RS); from those, 48 trials were irrigated. The evaluations were carried out in 5 yr, between 2017 and 2021, resulting in 12 400 grain yield observations (bushels per acre – bu/ac). Spatial adjustment was conducted under a multi‐stage analysis framework (Smith *et al*., 2001; Möhring & Piepho, 2009), where best linear unbiased estimators (BLUE) were obtained by fitting yield as a function of genotypes as fixed, row‐column as random, and residuals parameterized with an AR1 × AR1 autoregressive covariance structure. Standardized BLUEs data are available in the Supporting Information Dataset S1.

**Fig. 1:**
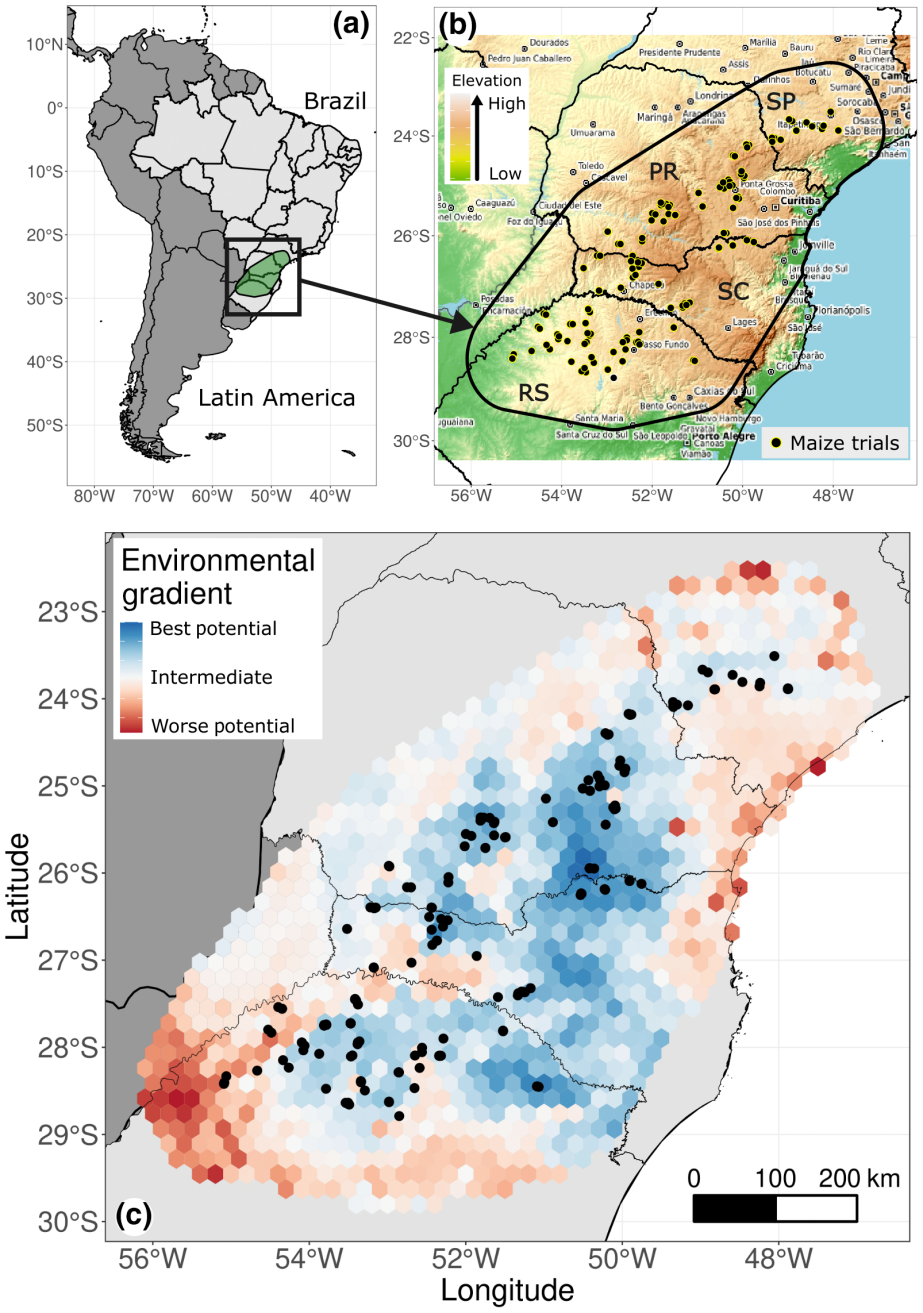
Description of the working area allocated in Latin America. (a) 183 experimental points with phenotypic data for maize were collected in an area covering the Brazilian states of São Paulo (SP), Paraná (PR), Santa Catarina (SC), and Rio Grande do Sul (RS). The texts shown in (b) of the figure are the names of municipalities distributed in these four states. The yield potential for the area is shown in (c), ranging from the best potential (blue) (i.e. regions capable of providing high grain yields), passing through an intermediate yield potential (white), to the lowest potential (i.e. low yield region, the red‐ish color).

The GIS environment was designed using the steps proposed by Resende *et al*. (2024). The study area was defined by interconnecting all outermost experimental points, generating a geoprocessing polygon, and later including a 50 km buffer beyond this polygon, as shown in Fig. 1(a,b). The pixel size (bin) was 25 km^2^ (5 × 5 km). Therefore, meeting these conditions, our prediction grid included 14 966 geographical bins. The datum was established following international standards using the WGS84 coordinate system.

### Envirotypic covariates

Environmental information included variables from four platforms: (1) MODIS satellite (https://modis.gsfc.nasa.gov/); (2) WorldClim (https://www.worldclim.org/); (3) NASA POWER (https://power.larc.nasa.gov/); and (4) SoilGrids (https://soilgrids.org/), returning a total of 1324 environmental covariates (MODIS = 803; WorldClim = 19; NASA POWER = 259; SoilGrids = 243). All envirotyping data were sourced using Python v.3.1 and the following packages: data from NASA POWER and WorldClim were downloaded using the requests package (pypi.org/project/requests); MODIS data were obtained with the pymodis package (pypi.org/project/pyModis); and SoilGrids data were accessed via the soilgrids package (pypi.org/project/soilgrids).

MODIS satellite images obtained for tile h13v11 from January 1, 2015, to December 31, 2021, were obtained at 16‐d intervals. NASA POWER data was collected for 1990–2019 and 2017–2019, the latter marking the latest data year. Bioclimatic variables from WorldClim included monthly temperature and rainfall, capturing annual trends, seasonality, and extreme conditions. Soil characteristics from SoilGrids, such as bulk density, cation exchange capacity, and organic carbon content, were downloaded for depths up to 200 cm, including mean values and 0.05, 0.5, and 0.95 percentiles.

### Development of Engineered Enviromic markers (EEM)

With the envirotyping data in hands, the enviromic markers were generated through the Random Forest (RF) model using Python's scikit‐learn package (pypi.org/project/scikit‐learn), analyzing yield from 183 trials across the study area. RF models, comprising N regression trees (function T), predict mean yield (m) based on environmental covariates (W), utilizing the CART algorithm and residual sum of squares for partitioning. Each tree, constructed from a random data subset (i∈I) with parameters (j∈J), contributes to the model's robustness. This process produced 10 000 predictors, with environmental means (m) derived from randomly selected genotypes to account for environmental variability's impact on genetic performance (Resende *et al*., 2021). The final enviromic marker was obtained by aggregating the predictors into seven distinct forests using hierarchical clustering. In summary, the $n^{\mathrm{th}}$ regression tree is fitted as:

$$m_{i\in I}=T_{n}\left(W_{i\in I,j\in J}\right),$$

and the $k^{\mathrm{th}}$ EEM ($\epsilon_{k}$) was generated by aggregating the similar predictors into a random forest defined as

$$\epsilon_{k}=N^{-1}\sum_{n=1,\;n\in k}^{N}T_{n\in k}\left(W\right).$$

Two strategies were used to validate the generation of EEM: correlation between observed and estimated values; and leave‐one‐out cross‐validation to evaluate the predictive capacity of the model for new environments bins not sampled. For the latter, a model was trained with the 182 trials to predict the 183^th^ and subsequently average the correlation between observed and predicted values. As a representative of all EEMs for charting visualizations, we constructed a single comprehensive marker, here referred to as ‘Wide‐Environment Gradient’, which includes all genotypes and all environmental covariates, also adjusted via RF.

### Reaction to Engineered Enviromic Markers (REEM) Model

Ensemble modeling involves combining predictions from multiple individual models to improve overall predictive ability. To model the responsiveness of genetic value to environmental factors, reaction norms for the set of EEMs were parameterized through the ensemble‐based random regression model (Schaeffer, 2004). The REEM model is linearly described as

y=Xb+Zu+e

where y denotes the vector of the response variable, X is the design matrix for fixed effects, and b is the vector of fixed effect coefficients, the fixed effects specifically include a unique intercept for each year, accommodating annual variations within the dataset; Z represents the matrix of random effects, which includes genetic terms and reaction norms, u is the vector of random effect coefficients, and e is the vector of residuals accounting for the nonexplained variability. Probabilistically, the model terms are described as:

$$y∼N\left(\mathrm{Xb},\mathrm{ZGZ}+R\right)$$

$$u∼N\left(0,G\right)$$

$$e∼N\left(0,R\right)$$

$$\mathrm{cov}\left(\mathrm{Zu},e\right)=0$$

where $R=I\sigma_{e}^{2}$ and $\sigma_{e}^{2}$ is the residual variance. The random effect corresponding to the genetics model term comprising overall genetic merit ($Z_{0}u_{0}$) and the set of K reaction norms ($Z_{k\in K}u_{k\in K}$) is decomposed as:

$$Z=\mathrm{BD}\left\{Z_{0},Z_{1},Z_{2},\ldots ,Z_{K}\right\}$$

$$u=\{u_{0},u_{1},u_{2},\ldots ,u_{K}$$

$$u∼N\left(0,\sum ⊗A\right)$$

further, Z consists of a block‐diagonal (BD) matrix $Z=Z_{0},Z_{1},Z_{2},\ldots ,Z_{K}$, where each block corresponds to a genotype (a first column of 1's, similar to traditional BLUP) and incorporates the random effects of the EEMs in the remaining columns. The vector $u=u_{0},u_{1},u_{2},\ldots ,u_{K}$ follows a multivariate normal distribution $u\sim N\left(0,\sum ⊗A\right)$, where *A* is the genetic relationship matrix, modeled using Wright's coefficients based on pedigree data to assess kinship, with dimensions equal to the total number of genotypes. The variance–covariance matrix ∑ has dimensions of $\left(K+1\right)\times \left(K+1\right)$, with the ij element describing the covariance between reaction norm terms $\mathrm{\sigma ij}=\text{cov}\left(u_{i},u_{j}\right)$. Here, K was set at seven EEMs, fueling the ensemble process for running the random regression model. In this setup, the model underwent 1000 runs, with each run capturing one of the enviromic markers previously grouped through hierarchical clustering.

The genetic correlations among bins were fed into Ward clustering method to define Breeding Zones, so that the genetic correlation is maximized within each zone. With this information, we projected the predicted yield of any given genotypes for all bins in the TPE grid into a map.

Stability coefficients were calculated under the assumption that the steeper the relationship between predicted yield for a given EEM, the more locally specialized the genetic material. Conversely, smaller reaction norm coefficients ($u_{1},\ldots ,u_{K}$) indicated more stable genotypes. The coefficients were transformed into angular degrees, so the closer to 0°, the more stable, and the closer to 45°, the more reactive. Negative coefficients are possible, as a genetic material may show a decrease in yield along a positive environmental gradient.

### Geo‐representativeness in genotypic recommendation

To enhance the predictive ability of selecting maize genotypes for each bin, we devised a comprehensive two‐step procedure. In the first step, genotypic ranking (Fig. S1A), we evaluated the genotypes in each bin. Using *t*‐statistical analysis, we identified the top‐ranked genotypes that were statistically tied with the best‐performing genotype. These statistically tied genotypes were then carried forward to the next phase of the selection process. In the second step, we introduced the concept of geographical representativeness (Fig. S1B) to further refine our genotype selection. We created multi‐maps of Euclidean distances based on local points containing repetitions of the genotypes under consideration. This procedure enabled us to assess the spatial distribution of each genotype and determine its representativeness within the geographical context. By analyzing the resulting overlaid maps, we were able to identify the genotype with the highest geographical representativeness for each bin. This selection criterion ensured that the chosen genotype not only performed well statistically but also had the greatest spatial coverage within the specific geographic area of interest.

### Enviromic prediction validations

To enhance the robustness of this study in the context of predicting genetic materials for untested environments or trials, we conducted cross‐validation of the models employing diverse strategies: The first approach involved inter‐state cross‐validation (SP, PR, SC, and RS). This entailed training the model on three states and validating it on the remaining fourth state (held out). Additionally, we employed the Leave‐One‐Out (LOO) strategy in the trials, whereby the model was trained on 182 of the utilized trials and used to predict the outcome for the remaining trial (183^rd^). To assess where the enviromics model performed well in the area, we conducted a kriging (i.e. spatial interpolation). Specifically, we fitted an ordinary kriging and theoretical variogram via a spherical (sph) model of the LOO predictive abilities as a function of latitude and longitude and plotted it on a heatmap. The procedure was carried out using the autoKrige function from the automap package in R (Hiemstra & Skoien, 2023).

In all the scenarios, two alternative models were employed. One was a baseline Genotype × Environment (G × E) model, accounting for irrigation effects as fixed and considering both genotypes and the G × E interaction, as random components, without any environmental covariates. The second was a Kernel‐type model. The methodology for the Kernel model was aligned with that outlined in the works by Jarquin *et al*. (2014) and Costa‐Neto *et al*. (2021b), albeit with the distinction that genomic data were not employed here, as described in our aforementioned models. It is pertinent to reiterate that this is a pure Enviromics study, devoid of amalgamation with other ‘omics. In the interest of comparative fairness, our Kernel matrix was constructed using the same Enviromic markers (Engineered Enviromic markers – EEM) as utilized in the random regression model outlined in this study. Both alternative models were executed using the sommer package in R (Covarrubias‐Pazaran, 2016).

The cross‐validation metrics used to assess predictive abilities included Pearson and Spearman correlations (between genotypic rankings) and Root Mean Squared Error (RMSE) for the observed vs predicted data by the models. Predictive abilities were evaluated separately for each trial to mitigate confounding effects potentially caused by Simpson's paradox (refer to Pearl, 2022 for more details). The assessment consistently applied metrics at the trial level, reflecting our focus on the models' capacity to accurately predict concealed genetic materials within each validation trial. Essentially, the analysis measured the models' ability to extrapolate and accurately predict genetic material rankings and values in environments not previously tested – evaluating their effectiveness in forecasting outcomes in new, unexplored locations.

## Results

In our analysis of genotype–by–management (G × M) interactions between irrigated and nonirrigated environmental conditions, we observed no clear differences, whether assessing broad‐sense (calculating genotypic BLUPs with total genetics) or narrow‐sense (calculating genotypic BLUPs with kinship additive genetics) relationships. While the broad‐sense interaction showed a slightly stronger trend, the difference was not conclusive. Although we observed some differences in yield levels, the relative rankings of the genotypes remained consistent across both conditions. More detailed information can be found in Fig. S2. Therefore, we chose to neglect this factor to simplify our analysis and focus on other important theoretical developments in Enviromics.

**Fig. 2:**
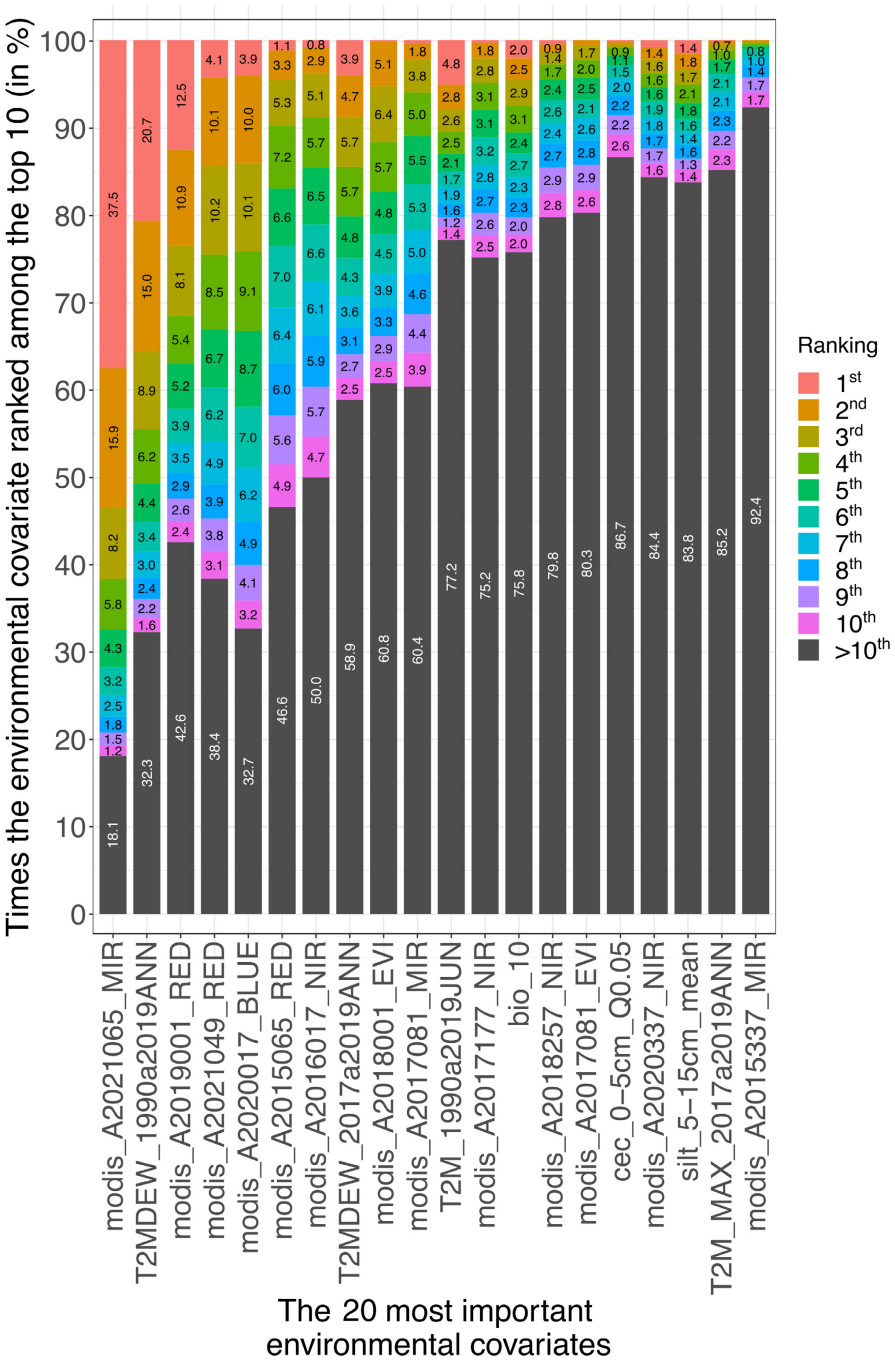
An enviromic‐wide association study depicting the importance of the top 20 most influential environmental covariates (*x*‐axis) in the Bootstrapping procedure for enviromic markers designing in *Zea mays* L. genotypes. The ranking level is shown, that is how many times (in %) a certain covariate was classified in 1^st^ place, 2^nd^ place, and so on (*y*‐axis).

After completing 1000 ensemble runs through bootstrapping procedure, we were able to quantify the significance of various environmental covariates on the expression of different genotype sets. Notably, MODIS data frequently emerged as highly significant, dominating the list of important variables in most analysis rounds. Specifically, out of the 1324 environmental covariates analyzed across 10 000 Random Forest model iterations, 225 covariates appeared at least once among the top five most important. Furthermore, 73 covariates ranked as the most important (top‐one) in at least one model iteration. Of these, the MODIS variables accounted for 37 instances, representing nearly 50% of the top‐one rankings. This prominent representation is reflective of MODIS constituting *c*. 60% of all the environmental covariates used, which could influence its prevalence in these significant positions. Fig. 2 provides a detailed overview of the results for the top 20 covariates, ranked according to their overall mean importance.

The covariate modis_A2021065_MIR (Middle Infrared) from 2021 ranked first in importance, followed by T2MDEW_1990a2019ANN from NASA POWER. In terms of environmental data sources, NASA POWER led with 33.88% importance, followed by WorldClim2 at 27.17%, MODIS satellite at 22.31%, and SoilGrids at 16.64%, totaling 100%. Alternative estimation methods like Multivariate Regression and Neural Networks (modeled using Python's scikit‐learn package) were explored; however, the Random Forest model proved to be more accurate based on the RMSE and the Pearson's correlation between predicted and observed data. Also, Random Forest efficiently handles high dimensionality and multicollinearity calibrates quickly and is less prone to overfitting (Belgiu & Drăguţ, 2016).

**Fig. 3:**
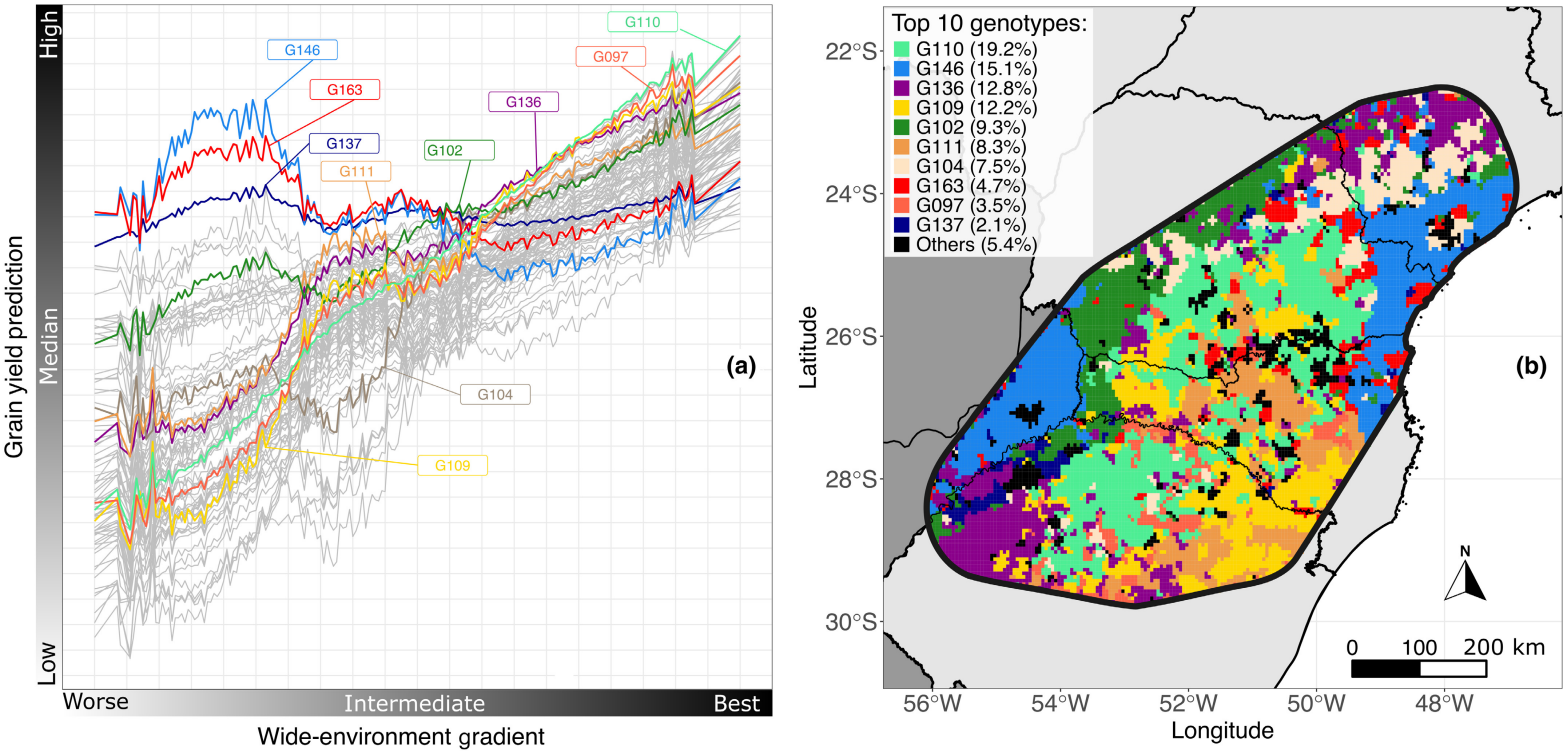
Behavior of the genotypic prediction along the environmental gradient in *Zea mays* L. hybrid genotypes. Only one enviromic marker is shown, but this result can be computed for all markers marginally. Part (a) shows the reaction norms; in part (b), we can follow the optimal recommendation of the top genotypes per bin for the whole area.

Fig. 3(a) illustrates the norm of reaction for genotypes, revealing an overall nonlinear pattern when considering the cumulative effects across all enviromic markers. However, when analyzed individually, each enviromic marker exhibits a linear response concerning its specific environmental parameters within the REEM model. This complexity arises from aggregating effects from all markers, suggesting variability in genotype performance across environments, while the response to each environmental factor remains straightforward and linear. Steeper lines denote greater adaptability (e.g. G104, G109), while flatter lines indicate stability (e.g. G137). Table 1 details adaptability/stability, derived from 10 K enviromic markers, with summary statistics presented. Fig. 3(b) ranks the top 10 genotypes by area occupation, showing the dominant genotype per bin and their area percentages, highlighting G110, G146, and G136 with 19.2%, 15.1%, and 12.8% occupation, respectively.

**Table 1:** Pedigree, representative metrics, the adaptability/stability coefficients (in angular degrees) per genotypes, and area occupation (in bins and percentage) after enviromics application.

Hybrid genotype^a^	Parents		Number of Bins with trials	Measured years	Adaptability/stability Coef. (°)			Area occupation (in bins and in %)
	Dam	Sire			Min.	Max.	Mean	
G110	G076	G035	176	2017, 2018, 2019, 2020, 2021	2.0	33.4	20.3	2873 (19.2%)
G146	G042	G016	34	2021	−11.5	11.0	−1.6	2260 (15.1%)
G136	G033	G042	102	2018, 2019, 2020	0.8	29.8	17.1	1916 (12.8%)
G109	G057	G079	71	2018, 2019	0.8	38.5	21.8	1826 (12.2%)
G102	G055	G080	125	2018, 2019, 2020, 2021	0.6	12.9	7.5	1392 (9.3%)
G111	G077	G035	41	2018	0.1	29.6	16.5	1242 (8.3%)
G104	–	–	63	2020, 2021	−0.4	21.2	10.0	1122 (7.5%)
G163	–	–	36	2021	−6.4	8.8	0.3	703 (4.7%)
G097	–	–	172	2017, 2018, 2019, 2020, 2021	1.7	37.4	22.4	524 (3.5%)
G137	G042	G044	54	2019, 2020	−2.6	3.8	0.7	314 (2.1%)

The TOP10 genotypes of the study area are displayed, which were the first ranked per bin. Dam is the female parent; Sire is the male parent. “–” indicates an unknown parent. It depicted the maximum, minimum, and mean yield values for the entire area of those genotypes. The adaptability/stability coefficient represents the maximum, minimum, and mean values for all 10 K enviromic markers.

^a^ All these genotypes were present in trials in the four Brazilian states (SP, PR, SC, and RS) and provided data from irrigated and nonirrigated trials.

**Fig. 4:**
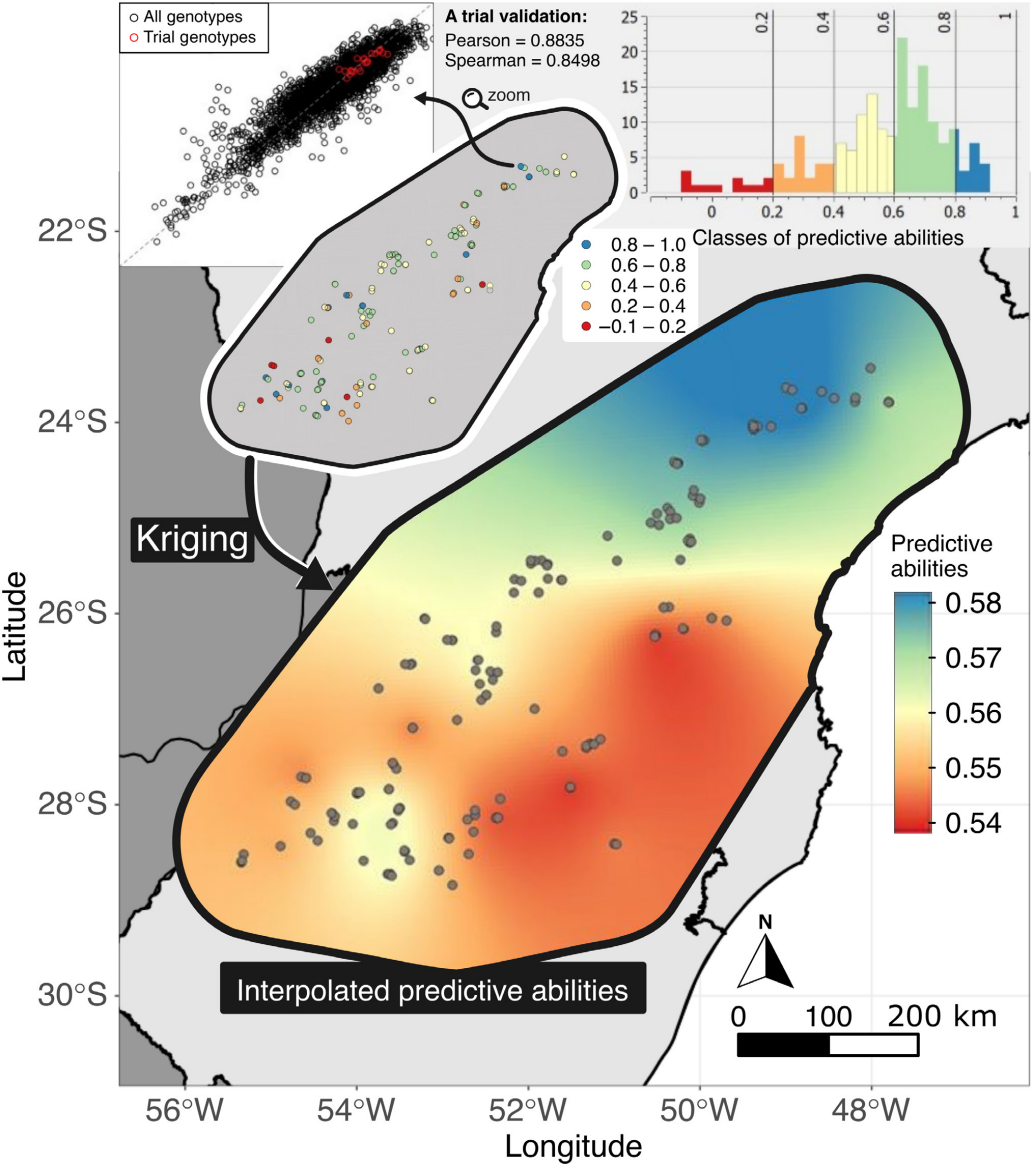
Visualization of the *Zea mays* L. genotypic predictive abilities in the work enviromics area. The main map shows spatial interpolation using Kriging, with colors indicating predictive strengths from 0.54 (red) to 0.58 (blue). The top‐left inset displays a cross‐validation scatter plot with Pearson and Spearman (ranking) correlation coefficients of 0.8835 and 0.8498, respectively, demonstrating a very‐successful case of genotypic trial prediction. Another view highlights the geographic distribution of trial sites, color‐coded by predictive ability classes from 0.0 to 1.0. The top‐right histogram categorizes these abilities, illustrating the distribution of genotypic performance across the region. The predictive abilities depicted in this chart are based on a leave‐one‐out cross‐validation procedure.

Table 2 evaluates the predictive abilities of three Genotype × Environment (G × E) model types – Baseline, Kernel, and Random Regression – using cross‐validation methods that include Spearman and Pearson correlations, and RMSE with its SE. These metrics were calculated for each state (PR, RS, SC, SP) and included total observation counts. Their performance was also analyzed using the Leave‐One‐Out (LOO) strategy. The outcomes quantify the models' predictive proficiency across different states and provide a comprehensive view of the analytical framework. Fig. 4 illustrates the spatial variation in predictive abilities via the Kriging procedure, showing consistent moderate to high genotypic performance across the region, supported by strong correlations from cross‐validation. The LOO results contributed to this Kriging analysis, and a successful trial prediction example is presented.

**Table 2:** Evaluation of predictive abilities using three G × E model types (baseline, kernel, and random regression).

Model	Baseline			Kernel			Random regression			*n*
State	Spearman	Pearson	RMSE	Spearman	Pearson	RMSE	Spearman	Pearson	RMSE	
PR	0.387 ± 0.24	0.408 ± 0.24	15.105 ± 13.80	0.418 ± 0.27	0.400 ± 0.26	15.089 ± 6.66	0.526 ± 0.20	0.540 ± 0.18	7.960 ± 4.44	62
RS	0.414 ± 0.27	0.438 ± 0.26	26.331 ± 44.91	0.371 ± 0.23	0.430 ± 0.23	18.110 ± 20.10	0.508 ± 0.23	0.549 ± 0.21	9.579 ± 13.85	69
SC	0.475 ± 0.26	0.479 ± 0.26	14.550 ± 9.89	0.456 ± 0.31	0.496 ± 0.32	15.377 ± 6.64	0.541 ± 0.23	0.545 ± 0.23	8.252 ± 4.54	34
SP	0.318 ± 0.20	0.328 ± 0.17	11.734 ± 6.13	0.453 ± 0.26	0.502 ± 0.25	14.098 ± 4.06	0.468 ± 0.17	0.474 ± 0.16	7.944 ± 2.78	18
Overall	0.407 ± 0.25	0.424 ± 0.25	18.903 ± 29.55	0.411 ± 0.25	0.439 ± 0.26	16.183 ± 12.81	0.516 ± 0.21	0.538 ± 0.20	8.623 ± 9.13	183
LOO	0.435 ± 0.25	0.449 ± 0.24	18.857 ± 29.36	0.447 ± 0.23	0.469 ± 0.23	17.111 ± 9.46	0.559 ± 0.21	0.579 ± 0.20	15.691 ± 8.84	183

Validation outcomes include Spearman correlations (ranking preservation), Pearson correlations, and RMSE (an indicator of model bias), with SE shown after the ‘±’ symbol. Validation schemes: SP (validation) using PR, SC, and RS (training); PR using SP, SC, and RS; SC using PR, SP, and RS; RS using PR, SC, and SP. ‘Overall’ merges predictions from SP, PR, SC, and RS into a single predictive ability. LOO, Leave‐One‐Out cross‐validation scheme includes 183 trials; RMSE, Root Mean Squared Error; *n* is the number of observations (trials) in the validation set.

The yield potential of the entire area is represented in Fig. 1, so the blue dots correspond to the highest yield potential considering the average of the genotypes. That is, it may be that an area marked with a blue dot is not good for a specific genotype. However, it is difficult to know whether the environment was good or bad for a genotype due to its characteristics *per se* or the management. Here we only included the information on whether the environment was irrigated.

**Fig. 5:**
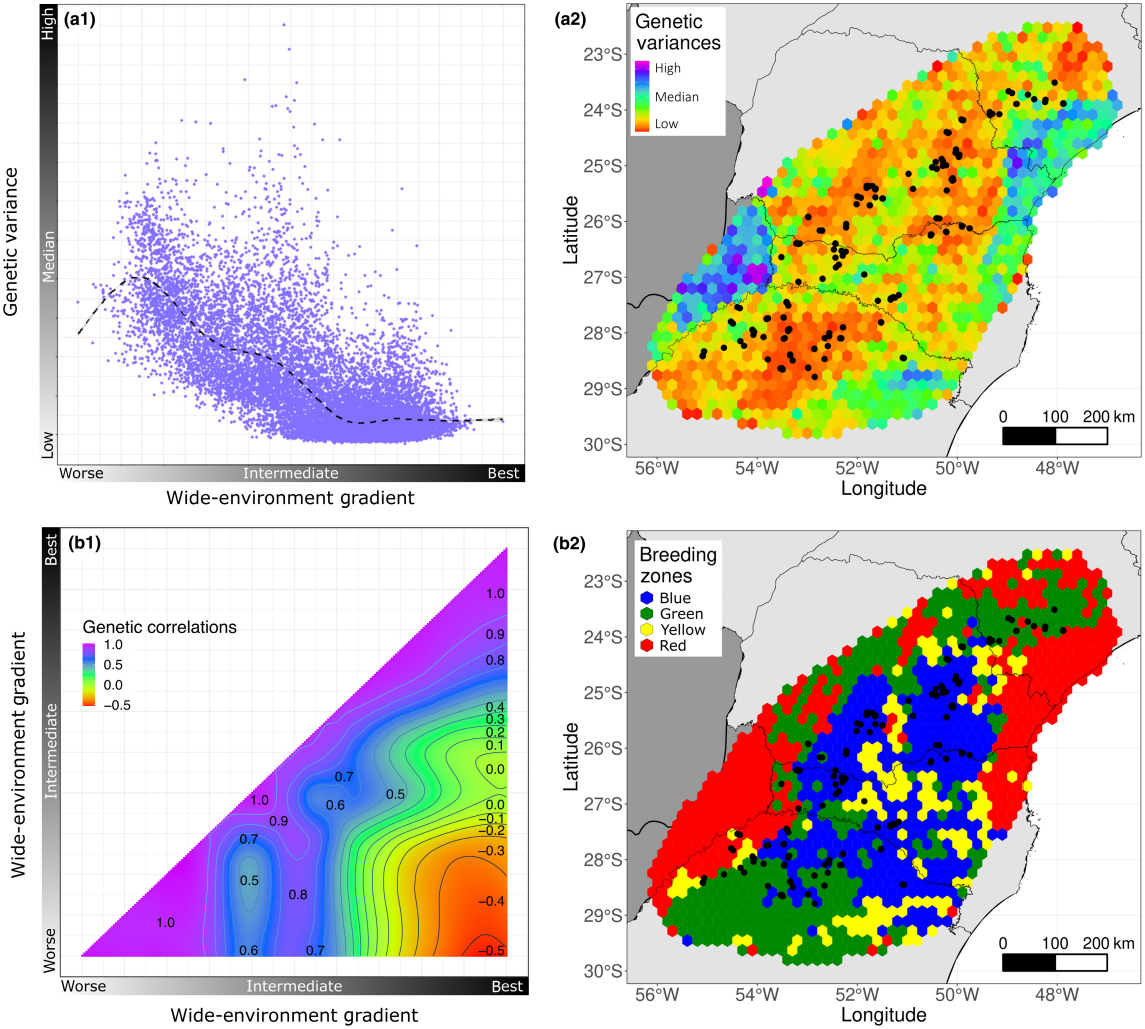
Genetic parameter studies for *Zea mays* L. breeding strategies. Parts a_1_ and a_2_ show an understanding of the distribution of genetic variances for the area, both along the environmental gradient of one of the enviromic markers (a_1_) and along the map longitudes and latitudes (a_2_). Parts b_1_ and b_2_ show an understanding of the distribution of genetic correlations for the area, both along the environmental gradient of an enviromic marker (b_1_) and by showing the breeding zones on the map longitudes and latitudes (b_2_). To a better clarification, bins were grouped in a hexagonal map.

Results of the analysis of genetic variation and correlation across different agricultural environments are summarized in Fig. 5 in four parts (a_1_, a_2_, b_1_, and b_2_). Parts a_1_ and a_2_ depict the additive genetic variation and their loess trend along the environmental gradient, showing higher variation in lower‐yield sites and lower in higher‐yield environments, with spatialized results for the study area in a_2_. Fig. 5(b_1_,b_2_) focuses on genetic correlation and the allocation of breeding zones to minimize G × E interactions. b_1_ displays a heatmap of genetically correlated values across environmental gradients, and b_2_ uses WARD clustering to group environments into four breeding zones (Blue, Green, Yellow, Red) based on environmental similarity and G × E analysis, optimizing genetic correlation within zones for consistent genotypic rankings.

Table 3 showcases results for four breeding zones: Blue, Green, Yellow, and Red, covering yield potential, top‐performing genotype yield, selection gain, bin, and trial count. For easy identification and to provide additional information, we have arranged/indicated the breeding zones in order from highest to lowest productivity. Yield potential varied, with Blue and Red colors representing the highest and lowest zones. The Red zone's top genotype had the highest selection gain, notably an increase of 22.36% by planting the top genotype. The genetic correlations within and between zones were significant, suggesting further evaluation in the Yellow zone. This table offers a detailed view of each zone's yield and genetic dynamics.

**Table 3:** Summary of the four Breeding Zones, referred to as ‘Blue’, ‘Green’, ‘Yellow’, and ‘Red’.

Breeding Zones	TOP1 Sel. gain^a^ (%)	Bin count	Trial count	Genetic correlations within and between Breeding Zones^b^			
				Blue	Green	Yellow	Red
Blue	8.65	3880	97	0.9286	0.9105	0.43314	0.0488
Green	8.96	4520	52	–	0.9299	0.6479	0.4142
Yellow	15.53	2417	3	–	–	0.9554	0.6166
Red	22.36	4149	31	–	–	–	0.8981

^a^ Selection gain (in %) of the TOP1 hybrid genotypes (per bin) in relation to all genotypes.

^b^ These genetic correlations are quantitatively described across the entire environmental gradient in Fig. 5(b_1_).

**Fig. 6:**
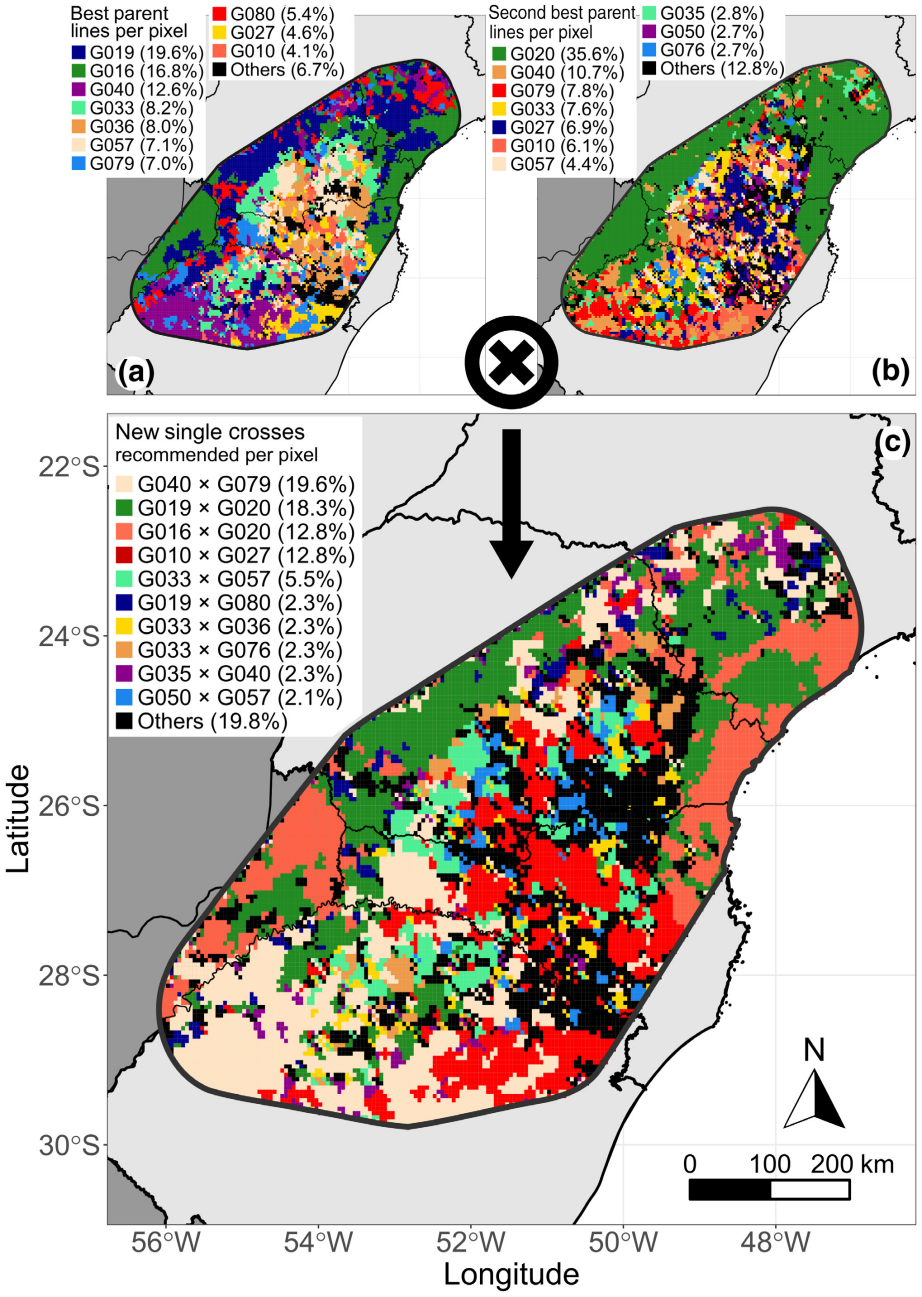
Maps of recommended parental lines for new maize hybrids compositions. Parts a and b show the parental lines better ranked by bin (or pixel). In part (a), the top‐ranked parental lines are presented, and in part (b), the second‐ranked parental lines are presented. Part (c) shows the combination of the parental line recommendations from parts (a and b), suggesting possible combinations for the formation of new hybrids. The highlighted area represents the region of interest for maize production.

The maps of recommended parental lines (G001–G085) for new maize hybrid compositions were generated to suggest possible heterotic combinations. Fig. 6(a) presents the top‐ranked parental lines by bin, while Fig. 6(b) presents the second‐ranked parental lines by bin. In Fig. 6(c), the combinations of Fig. 5(a,b) are shown, that is parental line recommendations suggesting new untested hybridizations.

## Discussion

### Importance of environmental covariates in the expression of enviromic markers, a preliminary study from the Envirome‐Wide Association Study (EWAS)

Environmental covariates significantly influence crop phenotypic expression, as shown in our Envirome‐Wide Association Study (EWAS) and detailed in Piepho (2022) and Costa‐Neto *et al*. (2023). Multiple environmental factors can shape maize yield and G × E interactions. The Random Forest importance metric, assessing the impact of each variable on model predictions, identified MODIS variables as particularly influential. This metric underscores each variable's significance in developing Engineered Enviromic Markers (EEM). Although MODIS was key in EEM development (see the top 20 covariates in Fig. 2), contributions to the Reaction to Engineered Enviromic Markers (REEM) model were distributed among data sources: NASA POWER (33.88%), WorldClim2 (27.17%), MODIS (22.31%), and SoilGrids (16.64%). These findings highlight the balanced role of each information source, emphasizing climatic variables. REEM uses EEMs to assess genotype yield potential and G × E interactions, supporting Dhillon *et al*. (2020). The importance of environmental covariates varies by local conditions and maize genotype, indicating the need for further research.

The environmental covariates collected from platforms like MODIS, WorldClim, SoilGrids, and NASA Power are instrumental in shaping maize yield because these influence various essential factors for plant growth (McClelland *et al*., 2021). MODIS tracks vegetation cover and surface temperature, impacting photosynthesis and transpiration. WorldClim provides weather data affecting yields, while SoilGrids informs about soil fertility, which is paramount for growth. NASA Power offers insights into solar radiation and temperature, influencing photosynthesis and evapotranspiration rates. Leveraging these technologies aids in understanding G × E interactions, enabling breeders to select adapted genotypes and optimize maize yield. Advanced statistical models highlight genetics' central role in growth under diverse conditions, stressing the importance of integrating genetic and environmental data for yield optimization (Adak *et al*., 2024a). Adak *et al*. (2024b) also reveal that the integration of enviromic and genomic data helped accurately predict maize flowering times, especially in locations with extended photoperiods, underscoring the direct influence of day length and solar radiation on flowering rates during critical intervals.

Some environmental conditions influencing maize yield potential and G × E interaction complexity are captured by the ‘modis_A2021065_MIR’ (MODIS) and ‘T2MDEW_1990a2019ANN’ (NASA POWER) variables, as shown in Fig. 2. Information on average infrared radiation from ‘modis_A2021065_MIR’ relates directly to surface temperature, affecting maize growth and development. Studies by Ban *et al*. (2019) and Sakamoto (2020) utilized MODIS covariates for predicting yields in maize and soybean, highlighting the importance of spectral radiation data. Wu *et al*. (2024) identify key leaf‐level photosynthetic traits influencing crop‐level Radiation Use Efficiency (RUE) in elite maize and grain sorghum hybrids, aiding in understanding how environmental covariates impact maize yield. Meanwhile, ‘T2MDEW_1990a2019ANN’ sheds light on air temperature and humidity. However, Duarte & Sentelhas (2020) note the importance of further research to clarify these variables' effects on maize yield expression, as their significance can vary with environmental conditions and genotypes.

Predictive modeling often favors variables with high covariance over direct causal relationships, recognizing that covariance does not equate to causality but can enhance predictive accuracy by providing additional information that causal variables may overlook (Breiman, 2001). In genomics, linkage disequilibrium (LD) among markers signifies genetic transmission rather than causality with the phenotype, necessitating consideration of LD, causality, and biological relevance in marker selection for accurate predictions (Resende, 2024). In genomic models, markers or covariates indirectly related to a trait can be effective predictors, highlighting the need to analyze a broad range of envirotypic variables for robust models (Resende *et al*., 2021; Costa‐Neto *et al*., 2023). Leveraging past lessons, transitioning from QTL‐mapping to embracing full genomic variations through GS models is now seen as key to advancing genomic research. Such strategies, including high‐throughput envirotyping, are essential for developing high‐yielding hybrids and are recommended for their predictive benefits in understanding complex environmental interactions (Resende *et al*., 2024).

This study assumes a prominent role by employing environmental covariates on a legitimate omic scale, underpinning the proposition of the term ‘Enviromics’ (Environmental + Omics) (Resende *et al*., 2021). Multi‐variable selection criteria, based on high‐throughput spectral images, are essential for assessing and selecting stress‐tolerant genotypes (Guadarrama‐Escobar *et al*., 2024). A total of 1324 environmental variables were employed; and, considering advancements in GIS, the integration of various other envirotyping platforms is feasible, such as the multispectral optical sensors or through approaches utilizing radar, thermal, or even LiDAR sensors (Newman & Furbank, 2021; Resende *et al*., 2024). Concurrently, Wulder *et al*. (2022) emphasized the central role of the Landsat program in scientific progress and terrestrial observation applications, with continuous spectral observations shaping significant transformations across various domains. Furthermore, Velastegui‐Montoya *et al*. (2023) unveiled, through bibliometric analysis, the increasing relevance of the Google Earth Engine platform in academic and scientific research, propelled by cloud‐based geospatial processing and its multifaceted applicability.

### Analysis of reaction norms in different agricultural environments

G × E could not occur between environmentally distinct locations but can be omnipresent even in site‐localized studies, highlighting the need for systematic examination of its dynamics in maize breeding to identify genotypes with superior stability and adaptability (Fritsche‐Neto *et al*., 2021). This task also enhances the importance of integrating environmental and genetic factors at an ‘omic’ level (Resende *et al*., 2021), aiming for a holistic strategy in maize breeding for optimal results. The cross‐validation results of the Baseline, Kernel, and ensemble‐based Random Regression models, shown in detailed metrics such as Spearman and Pearson correlations along with Root Mean Squared Error (RMSE), are reported (Table 2). Where Kernel models, recognized for their effectiveness in multi‐environment maize prediction (Bandeira e Sousa *et al*., 2017), displayed RMSE values ranging from 8.623 to 18.903.

The Random Regression model outperformed the others in correlation coefficients, with Spearman's scores from 0.541 for SC to 0.468 for SP, and Pearson's from 0.474 for SP to 0.549 for RS. These correlations (Spearman for genotypic ranking consistency and Pearson combined with RMSE for magnitude accuracy) underscore the models' predictive abilities. These values are encouraging, especially when considering that the prediction extends to an entirely untested region beyond the scope of the original training data. This type of prediction, which involves complex G × E, remains as a significant challenge in the field. Despite these difficulties, similar performance metrics have been reported by authors using advanced Bayesian models and AI techniques, underscoring the robustness of these methods across diverse datasets (Xavier, 2021; Araújo *et al*., 2024).

The Baseline and Kernel models showed moderate validation performance, with Spearman correlations between 0.318 to 0.475 and Pearson correlations from 0.328 to 0.502, with the Kernel model slightly outperforming the Baseline model. Concerningly, Kernel‐based Enviromic models add directly the similarity between environments, not the genetic similarity between trials; therefore, more information about this genetic covariance is needed (Gevartosky *et al*., 2023). The Random Regression model excelled in computational efficiency and capability to extensively predict across the studied area, effectively integrating various environmental variables. By contrast, the Kernel models were slower and struggled with pixel‐by‐pixel prediction, failing to capture genetic covariance, whereas the Baseline model was noted for its computational speed and simplicity despite not using environmental covariates. Overall, the ensemble‐based Random Regression model offers an optimal combo of predictive ability and efficiency, it is promising for integrating environmental variables and future research.

Understanding G × E interactions across broad geographic areas requires data from multiple locations, but breeding experiments aren't always feasible everywhere (Marcatti *et al*., 2017). In such cases, unbalanced or single‐genotype data from on‐farm records by local producers can serve as a valuable alternative, despite being less controlled. These real‐world cultivation data enhance phenotypic value reliability, offering insights into G × E patterns across diverse environments (Schmidt *et al*., 2018). This study benefited from experimental data provided by the company, potentially aligning genotype recommendations more closely with breeding test conditions rather than solely relying on field realities.

The prediction presented in Fig. 3 was obtained considering that an experiment had been conducted in each of the 14 966 bins. From this result, we can simply choose which genotype will be the most appropriate for the property located in a given bin. However, we also added to this choice a metric of representativeness of the hybrid genotype in the location. If a certain genotype has a high predicted yield for a location but does not present many occurrences of experimental data in the region, this should trigger an alert to the breeder. This genotype should be replaced by another with statistically equivalent yield but with a greater local representation.

A good indication that our representativeness metric is effective is that all genotypes selected for the area were measured in the four Brazilian states (SP, PR, SC, and RS) and also in trials with and without irrigation (see Table 1). Even so, the genotypes G146, G111, and G163 presented only one year of measurement, and therefore, if appropriate the breeder could choose other genotypes (second and third place for the bin) if they do not present the confidence needed for a recommendation. From another selection perspective, as our analyses used data up to 2021, validating our results today, some of the GIS‐based evaluated hybrids have become commercial cultivars (results omitted due to company confidentiality).

Choosing between more adaptive or stable genotypes depends on environmental variability, breeding goals, and desired traits (Resende *et al*., 2018). Adaptive hybrids such as G104 and G109 are favored in variable environments due to their higher adaptability and responsiveness. Conversely, in stable environments, hybrids are less affected by variations and show consistent performance, such as G137 is preferred. Breeding objectives, whether aiming for higher yields or stability, also guide hybrid selection. Additionally, as our single‐trait analysis focused on grain productivity, traits like disease resistance and grain quality should also be considered in practice. Some high‐performing genotypes cannot be released as cultivars due to evaluations of other traits. Thus, evaluating hybrids across various conditions and goals is essential before deciding and planning.

### Addressing spatial distribution challenges, breeding zones, and genetic variances in maize TPE

Within the spatial distribution of our trial locations (Fig. 1c), points concentrate in regions with the ‘best’ yield potential (blue areas). This raises concerns about the representativeness of our training dataset and its impact on the model's predictive ability across different conditions. Our models assess yield potential from ‘best’ to ‘worst’ (blue to red areas). However, these worst locations are less relevant as maize plantings traditionally concentrate in climatically favorable areas. The yield potential map shows high potential areas may not be optimal for every genotype, highlighting the complexity of G × E interactions. Additionally, Fig. 4 shows that worse locations do not always have the lowest predictive abilities. To improve validation and understand genotype performance under varied conditions, we recommend expanding trials into lower‐yield areas, especially those with sparse data (Fig. 5b_2_). Increasing experimental plots in ‘worse’ potential areas (red) will enhance the model's predictive power and ensure more accurate, comprehensive maize yield predictions.

Selecting experimental locations to maximize genetic variance is also key for identifying high‐performing genotypes (Prus & Piepho, 2021). Conversely, experiments in areas lacking the potential for genetic variance expression may lead to inefficient genotype differentiation. It is pivotal to select regions inducing genetic variability, allowing genotypes to fully express their phenotypic potential and facilitating the identification of superior genotypes. For instance, maize experiments across varied rainfall, soil, and altitude conditions can enhance environmental variation and the chances of detecting superior genotypes. Interestingly, areas with lower‐yield potential showed greater genetic variances than those with higher‐yield potential (Fig. 5a_2_). It suggests that excessive experimental controls might not effectively reveal G × E interaction, potentially leading to inefficient selection. Similar results were observed by Resende *et al*. (2018) when assessing eucalyptus hybrids genotypes.

Even similar environments can exhibit different G × E interactions, shaping the genotype's phenotype uniquely. Oftentimes, grouping locations by overall environmental similarities into mega‐environments might not accurately control G × E interactions (González‐Barrios *et al*., 2019; Callister *et al*., 2024). Hence, this suggests that a more effective strategy could be grouping by G × E interactions linked to enviromic markers, and thus genetic covariances between environments. The adopted method focusing on G × E rather than environmental traits, better defines breeding zones. Consequently, four zones (Blue, Green, Yellow, and Red) were established based on G × E patterns (Table 3), each with unique genetic correlations and yield potentials. The Blue zone showed the highest average yield and strong genetic linkage with the Green zone, whereas the Red zone, despite the highest TOP1 selection gain, had the lowest genetic correlation with other zones (Fig. 5b_2_).

### Enviromics in maize breeding: recommending parental lines for new maize hybrid compositions

To increase the probability of obtaining improved hybrids, breeders cross divergent inbred lines expecting to explore heterosis. These lines are frequently recycled to produce new elite lines, aiming to generate elite lines with a high frequency of favorable alleles. From a limited number of lines, it is possible to obtain a very large number of hybrids, making field evaluations unfeasible. (Mushayi *et al*., 2020; Fritsche‐Neto *et al*., 2021). Also, in general, maize breeding programs seem to exploit low levels of genetic diversity. A limiting factor is to perform accurate high‐throughput phenotyping for the comprehensive and efficient characterization of maize germplasm for key traits (Prasanna, 2012). In this sense, the use of environmental covariates may add important information to help increase the efficiency of breeding programs.

The recommendation of untested genotypes in a specific area offers several advantages when based on environmental models (Resende *et al*., 2022). First, it maximizes genetic potential, enhancing yield by aligning genotype adaptability with specific environmental conditions. Second, it saves time and resources by obviating the need for extensive tests across various locations to assess adaptability. Third, it accelerates genetic improvement response to market needs and changing environments by swiftly adapting genotypes to new areas based on environmental models. Furthermore, such recommendations mitigate the risk of the adaptation process, as models consider diverse environmental and genetic variables influencing variety performance in a given area (Costa‐Neto *et al*., 2021b). However, the field validation of these recommendations remains essential before large‐scale implementation.

In this study, we predict the behavior of the parents (inbred lines) based on the phenotypic information of their hybrids. For that, we focused on additive effects; however, nonadditive effects, with the dominance that provides heterosis, would be very welcome here. Since it was not the focus of this study to study these, we did not aim to saturate the results. In Fig. 6 (parts a and b), it is possible to see how the recommendation of parental lines for the considered region would look. The best lines placed per bin are in Fig. 6(a), and the second‐best lines are in Fig. 6(b). In Fig. 6(c), we suggest the crossing between the first‐ and second‐best lines, forming a new local hybrid combination.

Developing site‐specific hybrids is a major challenge in maize genetic breeding (Mushayi *et al*., 2020), since a complete breeding program is needed to develop parent lines adapted to specific regions. The process involves selecting desirable traits through several generations of self‐fertilization, which requires a significant amount of time and resources. In addition, the crossing process to form hybrids is also complex and laborious. The success in obtaining site‐specific hybrids depends on the identification of parent lines that present a favorable interaction with the targeted environment and requires the evaluation of plant performance in different locations (Fritsche‐Neto *et al*., 2021; Kusmec *et al*., 2023). This makes the process of developing specific hybrids a challenge that requires a lot of effort, time, and resources.

Given the global cultivation of maize across various latitudes and longitudes (Ortiz *et al*., 2010), enviromics play a differential role in maize genetic breeding by building markers linked to genotype performance in diverse environments. These markers aid in pinpointing areas of adaptation for developing well‐suited cultivars for specific macroenvironments, enhancing genetic improvement efficiency. Enviromic markers facilitate the creation of locally tailored hybrids, boosting maize yield for particular geographic regions. Moreover, enviromics expedites the early selection of promising genotypes, streamlining genetic improvement and reducing field evaluation costs. Overall, this approach enhances the selection process efficiency in maize breeding, enabling breeders to develop cultivars thriving in diverse environments, crucial for modern agriculture success.

### Current and future directions in crop breeding enhanced by enviromic marker engineering

Exploring the potential of enviromic markers is a promising research avenue, with Envirome‐Wide Association Studies set to examine multi‐environmental factors beyond the current scope (Piepho, 2022; Costa‐Neto *et al*., 2023). This may involve integrating alternative data sources, such as remote sensing data, or developing new satellite techniques for extracting information from existing datasets (Newman & Furbank, 2021; Resende *et al*., 2024). These advancements promise to deepen our understanding of crop‐environment interactions, enhance efficiency, and improve resource allocation in genetic improvement (Gevartosky *et al*., 2023). The growing global demand for agricultural goods necessitates accelerated crop improvement methods. High‐throughput genomic, enviromic, phenomic (G–E–P), and other multi‐omic data collection methods have overcome previous data acquisition bottlenecks (Resende *et al*., 2021). However, leveraging large, high‐dimensional datasets remains a challenge, where AI emerges as a promising solution (Xu *et al*., 2022; Negus *et al*., 2024).

Field experiments validate the practical value of recommended parental lines and hybrids, highlighting areas for enhancement and confirming their real‐world applicability (Bernardo, 2021). Using environmental covariates along with kinship data can optimize the development of site‐specific hybrids, thereby improving crop performance under stress conditions (Pérez‐Rodríguez *et al*., 2015). Refining models to more comprehensively capture the complexities of heterosis, including the incorporation of genetic dominant effects and leveraging research on drought and heat tolerance. For example, the study by Kusmec *et al*. (2023), enables researchers to better guide crop development adapted to climate change. Further validation of these models through targeted on‐field experiments could reveal additional refinement opportunities. This can enhance the practical utility of the developed parental lines and hybrids ensuring these meet the sophisticated demands of modern agriculture (Ferrão *et al*., 2020).

In enviromics, generating reliable genotypic predictions is the gateway to ensuring high phenotypic performance. According to Resende *et al*. (2024), the ability to make accurate predictions is more important than explaining how each environmental covariate affects phenotypic traits. Therefore, rigorous validation of predictive models is necessary. In this study, we used two robust cross‐validation methods: leave‐one‐out (LOO) and leave‐out‐regional‐samples. LOO is popular and serves as a reference in validation. The leave‐out‐regional‐samples method minimizes prediction contamination from spatial dependency, preventing overestimation of predictive ability. Other methods, such as leave‐out‐new‐samples, leave‐out‐environments, leave‐out‐genotypes, leave‐out‐year, and bidirectional methods, can also evaluate model performance (Rogers & Holland, 2022). In future work, we plan to include leave‐out‐year and bidirectional leave‐out‐year + leave‐out‐regional‐samples methods in our validation pipeline to further ensure good model performance and achieve high predictive ability for traits such as yield potential and disease resistance.

Looking ahead, integrating data from controlled experiments and real‐world field conditions offers a promising way to refine agricultural research. Expanding this research to other crops is an intriguing prospect. The methodologies and findings of this study could be adapted to diverse crop varieties, transcending the maize domain. This research highlights the feasibility of conducting breeding ‘*virtual trials*’ (Resende *et al*., 2024), bypassing the need for physical experiments or commercial stands when datasets are integrated into enviromics models. This method could save significant resources by eliminating the need for soil preparation, fertilization, planting, harvesting, and phenotyping. Impressively, the predictive ability of these virtual trials can approach 90% compared to traditional field trials. Analyzing the interplay between genetic and environmental elements in different crops would provide a comprehensive understanding of optimizing crop yield across varying circumstances.

## Competing interests

None declared.

## Author contributions

RTR, AX, PITS and GEM designed the study. RTR and GEM led the project, conducted data analyses, developed figures and tables and wrote the manuscript. AX led cross‐validation protocols, maintained manuscript quality and contributed to drafting. PITS managed data, performed data mining and contributed to manuscript development. MPMR provided insights on maize improvement and contributed to manuscript development. DJ validated analyses and contributed to manuscript development. All authors discussed the results, revised and agreed on the final version of the manuscript. RTR and GEM contributed equally to this work.

## Supporting information


**Dataset S1** Phenotypic data.

**Fig. S1** Algorithm for genotype selection per bin based on two metrics: genotypic ranking and geographical representativeness.
**Fig. S2** Scatter plot showing genotypic values under irrigated and nonirrigated conditions.
Please note: Wiley is not responsible for the content or functionality of any Supporting Information supplied by the authors. Any queries (other than missing material) should be directed to the *New Phytologist* Central Office.

## Data Availability

The data that supports the findings of this study are available in Dataset S1.
