# Supplementary material for: GIS‐based G × E modeling of maize hybrids through enviromic markers engineering
Source: New Phytol. 2024 Jul 16;245(1):102–16. doi: 10.1111/nph.19951 (PMC11617650; doi:10.1111/nph.19951)
Supplement: Supplementary file 2 — Fig. S1 Algorithm for genotype selection per bin based on two metrics: genotypic ranking and geographical representativeness. Fig. S2 Scatter plot showing genotypic values under irrigated and nonirrigated conditions. Please note: Wiley is not responsible for the content or functionality of any Supporting Information supplied by the authors. Any queries (other than missing material) should be directed to the New Phytologist Central Office. [file NPH-245-102-s002.pdf]

Article title: GIS-based G×E Modeling of Maize Hybrids through Enviromic Markers Engineering.

Authors: Rafael T. Resende; Alencar Xavier; Pedro Italo T. Silva; Marcela P. M. Resende; Diego Jarquin; Gustavo E. Marcatti.

Article acceptance date: 22 June 2024.

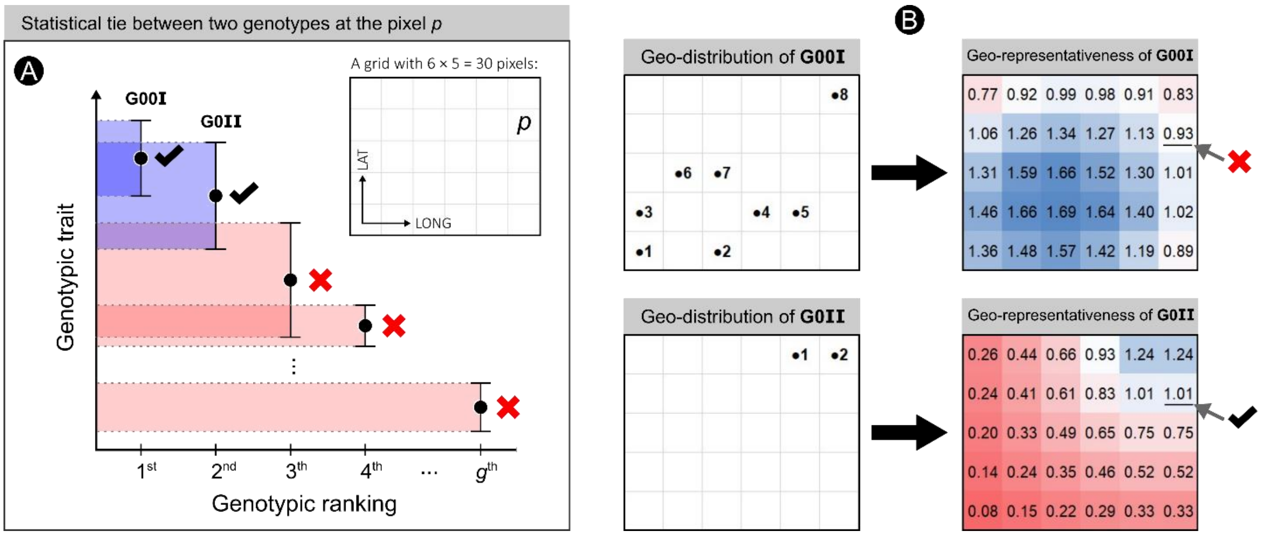

Figure S1. Algorithm for genotype selection per bin based on two sequential metrics: genotypic ranking (part A) and geographical representativeness (part B). In part A, at the pixel (or bin) 'p', the genotypes statistically tied for the best rank (G00I and G0II) proceed to the next phase. In part B, a consistency procedure is presented where the selected genotype is determined based on the highest geographical representativeness, obtained by overlaying Euclidean distance maps for each point containing a repetition of the genotype within the grid.

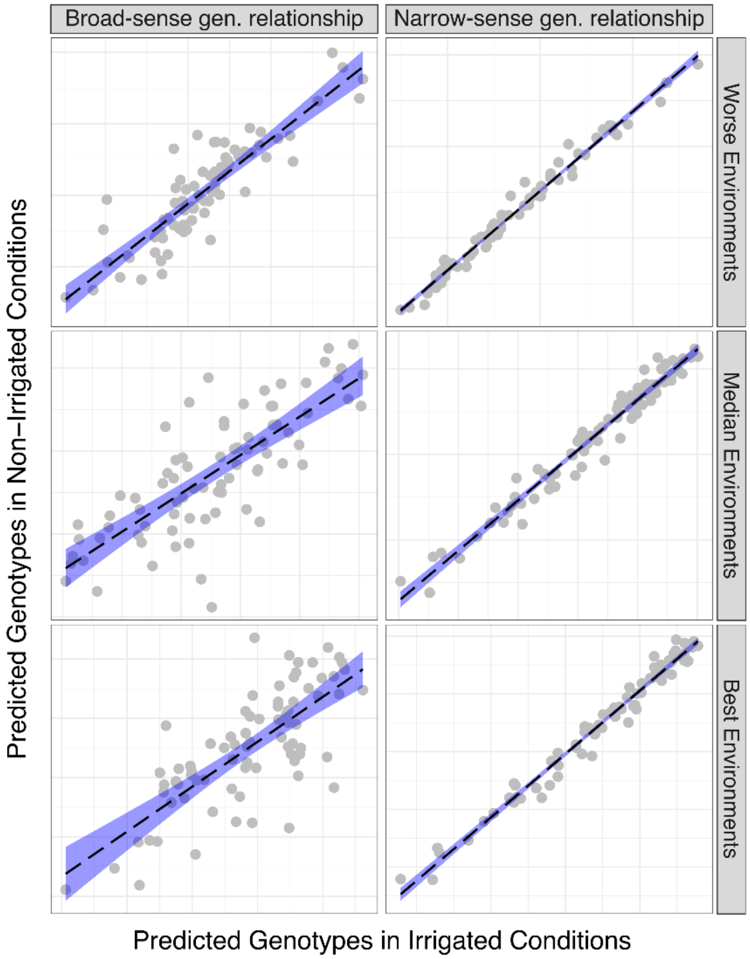

Figure S2. Scatter plot depicting six facet combinations. In all plots, the X-axis represents genotypic values under irrigated experimental conditions and the Y-axis represents non-irrigated genotypic values. The facets group adjusted genotypic additive values (breeding values or narrow-sense) and total genotypic values (or broad-sense) (columns), across three different environments (rows): low potential yield (Worse Environments), medium potential yield (Median Environments), and high potential yield (Best Environments). A dashed trend line indicates the linear relationship.
